# The Expression of Perilipin Family Proteins can be used as Diagnostic Markers of Liposarcoma and to Differentiate Subtypes

**DOI:** 10.7150/jca.41736

**Published:** 2020-04-07

**Authors:** Qiaochu Zhang, Pengpeng Zhang, Bingcheng Li, Hongwei Dang, Jinfang Jiang, Lian Meng, Haijun Zhang, Yangyang Zhang, Xiaomeng Wang, Qianru Li, Yang wang, Chunxia Liu, Feng Li

**Affiliations:** 1 Department of Pathology and Key Laboratory for Xinjiang Endemic and Ethnic Diseases, The First Affiliated Hospital, Shihezi University School of Medicine, Shihezi 832002, China; 2 Department of Pathology and Medical Research Center, Beijing Chaoyang Hospital, Capital Medical University, Beijing 100020, China; 3 Department of Pathology, Beijing Friendship Hospital, Capital Medical University, National Clinical Research Center for Digestive Diseases, Beijing, 100032 China

**Keywords:** Perilipin, Liposarcoma, Non-lipomatous sarcoma, Tissue microarray, Immunohistochemistry

## Abstract

**Objective**: Liposarcoma is a mesenchymal malignant tumor characterized by adipocyte differentiation which is divided into four subtypes with different prognosis. Accurate histopathological diagnosis is essential for precise treatment. Perilipins, including PLIN1, PLIN2, PLIN3, PLIN4, PLIN5, is a family of lipid droplet-associated proteins that participate in lipid metabolism regulation. The role that perilipins play in sarcomas is not clear. This study aims to assess perilipins expression in subtypes of liposarcoma and various non-lipomatous sarcomas.

**Methods**: A large set of 245 soft tissue sarcoma paraffin-embedded samples including 66 liposarcomas and 179 non-lipomatous sarcomas were collected for tissue microarray and immunohistochemistry to assess perilipins expression.

**Results**: PLIN1 expression was shown in most liposarcomas (41/66) and was absent in non-lipomatous sarcomas (0/179). PLIN4 expression was shown in some liposarcomas (21/66) and was almost negative in non-lipomatous sarcomas (2/179). PLIN1 and PLIN4 expressions in liposarcoma were higher (both P<0.001) than those in non-lipomatous sarcoma. Both PLIN1 and PLIN4 also had a significant difference in liposarcoma subtypes (both P<0.001). PLIN2, PLIN3 and PLIN5 were widely expressed in liposarcomas, rhabdomyosarcomas, leiomyosarcomas, dermatofibrosarcoma protuberans, undifferentiated sarcomas, fibrosarcomas, Ewing's sarcomas and epithelioid sarcomas. PLIN2, PLIN3 and PLIN5 expressions were significantly different among non-lipomatous sarcoma (all P<0.01).

Except for PLIN3, the expression of the other four perilipin members in liposarcoma was pairwise related.

**Conclusions**: PLIN1 and PLIN4 can be used as diagnostic markers of liposarcoma and to differentiate liposarcoma subtypes. The combined application of whole perilipin family immunohistochemistry may help to distinguish differently differentiated sarcomas.

## Introduction

Liposarcoma is one of the most common sarcomas in adults, accounting for 24% and 45% 1 of limb and retroperitoneal soft tissue sarcomas, respectively. It occurs mostly in the lower extremities, retroperitoneum, mesentery, and shoulders. Liposarcoma originates from mesenchymal tissues in which lipoblasts differentiate into adipocytes. It is characterized by abnormal lipoblasts with different degrees of differentiation in tumors, such as primitive mesenchymal cells, fibroblast-like cells, lipoblasts of early, middle, and late stages, giant lipoblasts, and mature adipocytes. In 2013, the World Health Organization (WHO) classified liposarcoma into different subtypes, which are atypical liposarcoma (ALT), well-differentiated liposarcoma (WDL), dedifferentiated liposarcoma (DL), myxoid/round cell liposarcoma (ML/RCL), and pleomorphic liposarcoma (PL) 2, 3. As they present different prognoses, histopathological diagnosis is essential, and immunohistochemistry assists with making the diagnosis accurate 4.

Recent research has already shown that some immunohistochemical molecules can be helpful in diagnosing subtypes of liposarcoma. To name a few, S-100 can be used to mark lipoblasts in liposarcoma 5. Genetic alterations in ALT/WDL and DL include telomere fusion, ring chromosome, giant marker chromosome, and MDM2 and CDK4 amplification 6. In ML, the translocation of chromosome t (12; 16; q13; P11) leads to the fusion of DDIT3 and FUS, while t (12;22) causes the fusion of DDIT3 and EWSR1 7. Although the diagnosis of liposarcoma benefits from those variations, they do not have specificity in liposarcoma 8. The molecular-targeted therapy of soft tissue sarcoma has been widely explored 9, and the discovery of more specific diagnostic immunohistochemical markers for liposarcoma may help distinguish subtypes and provide ideas for targeted therapy.

The perilipin family 10 is a set of structural proteins embedded in the surface of lipid droplets. It maintains the stability of lipid droplets and participates in the regulation of lipid metabolism 11. Perilipins include perilipin (PLIN1), adipocyte differentiation related-protein or adipophilin (PLIN2) 12, 47 kDa tail-interacting protein (PLIN3) - also known as placental protein 17 or mannose 6 phosphate binding protein 1^13^, plasma membrane-associated protein (PLIN4), and myocardial lipid droplet protein, oxidative perilipin, or lipid storage droplet protein 5 (PLIN5). These are involved in the formation and transportation of lipid droplets 14. In lipid droplets development, small lipid droplets of early fat cells are mainly covered with PLIN2 and PLIN3, whereas large lipid droplets of mature fat cells are mainly covered with PLIN1. In adipocyte differentiation, the levels of PLIN2 decrease, whereas the levels of PLIN1 increase 15. The abnormal expression of perilipins is associated with the occurrence and development of various diseases, such as atherosclerotic disease 16, fatty liver 17, inflammation 18, sebaceous cancer 19, and gastrointestinal neoplasm 20. Recent research also reported the different expressions of some perilipin family proteins in some kinds of mesenchymal tumor and the increase of PLIN1 expression with adipocytic differentiation of liposarcoma 21, 22. However, the role that the whole perilipin family proteins play in various mesenchymal malignant tumors should be further investigated. In view of this, the purpose of our research is to observe the expression of perilipin family proteins in a large cohort of subtypes of liposarcoma and various non-lipomatous sarcoma by immunohistochemistry combined with microarray. The study also aims to explore the relationship between perilipins expression and clinical pathological parameters.

## Materials and methods

### Tissue sample selection

The pathology archives at The First Affiliated Hospital of Shihezi University School of Medicine between January 2010 and December 2017 were searched for a total of 245 cases, and 66 liposarcomas and 179 non-lipomatous sarcomas were included in this study. Among the 66 cases of liposarcoma, 14 ALT or WDL cases, 38 ML cases, 3 PL cases, and 11 DL cases were included. Among the 179 cases of non-lipomatous sarcoma, there were 22 rhabdomyosarcoma cases, 42 leiomyosarcoma cases, 38 dermatofibrosarcoma protuberans cases, 34 undifferentiated sarcoma cases, 18 Ewing's sarcoma cases, 18 fibrosarcoma cases, and 8 epithelioid sarcoma cases. A total of 20 samples of normal adipose tissue were also identified from the pathology archives as the control group. The H&E staining of each tumor type is exhibited in the supplementary figure (Figure S1). All of the histologic slides were reviewed and classified by experienced pathologists through immunohistochemistry and molecular detection in accordance with the WHO classification of tumors.

### Tissue microarray (TMA) construction

Tissue microarrays (TMAs) were constructed from paraffin-embedded tissue blocks from selected eight types of tumors that had sufficient tissue. A typical morphological area of soft tissue sarcoma was included in the TMA which included two 1.0mm cores taken from each tissue block to account for tumor heterogeneity. TMA blocks were selected by a trained pathologist. The Minicore instrument and TMA Designer 2 software were used to design and create tissue cores.

### Immunohistochemistry (IHC) staining and assessment

All paraffin-embedded tissue samples were cut into 4μm thick sections which were dewaxed in xylene and rehydrated using graded concentrations of ethanol solutions. After hybridization, antigen retrieval was performed using a citrate buffer solution (0.02mol/L; pH 6.0) in an autoclave at 100°C for eight minutes and cooled at room temperature. Endogenous peroxidase activity was blocked by incubating in a 3% peroxide-methanol solution for ten minutes. Afterwards, the sections were washed with phosphate-buffered saline (PBS) three times, for five minutes each time. Immunohistochemistry was performed on all cases separately, reacting with anti-plin1 antibody (rabbit monoclonal antibody, 1:400 dilution; Cell Signaling, China), anti-plin2 antibody (rabbit polyclonal antibody, 1:600 dilution; Proteintech, China), anti-plin3 antibody (mouse monoclonal antibody, 1:50 dilution; Santa Cruz Biotechnology, USA), anti-plin4 antibody (rabbit polyclonal antibody, 1:1,000 dilution; Novus Biologicals, USA), and anti-plin5 antibody (rabbit polyclonal antibody, 1:1,000 dilution; Novus Biologicals, USA). Human small intestine tissue served as positive control. Positivity was determined based on cytoplasmic or membranous staining for PLIN2, PLIN4 and PLIN5, membranous staining for PLIN1, and nuclear or cytoplasmic staining for PLIN3. The sections were incubated overnight with primary antibody at 4°C. The residual antibodies were replaced with PBS and incubated with secondary antibody (DAKO) for 40 minutes. The staining reaction was intensified with diaminobenzidine (DAB-Sigma). Slides were washed with water, then counterstained with hematoxylin, and dehydrated. Every case was independently scored at 40X magnification in at least five fields. Considering the heterogeneity of sarcoma, we obtained the immunohistochemical score by multiplying the percentage of tumor positive cells (score of 1: 0-5%, score of 2: 5.1-50%, score of 3: 50.1-80%, score of 4: 80.1-100%) by the intensity of staining (score of 0: negative, score of 1: weak, score of 2: moderate, score of 3: intense)^21^, ^23^. PLIN1 shows membrane staining, PLIN2, PLIN4 and PLIN5 exhibits cytoplasmic or membrane staining, and PLIN3 shows the nuclear or cytoplasmic staining pattern. The multiplication score indicated that a score of 0 or 1 was considered as negative expression (-), a score of 2-4 was considered as weakly positive expression (+), and both a score of 5-8 (++) and 9-12 (+++) were considered as intensively positive expression.

### Statistical Analysis

The differential expression of the perilipins in liposarcoma and non-lipomatous sarcoma and the relationship between the expression of perilipins and the clinical pathology parameters were determined through Chi-square, Fisher's exact or Kruskal-Wallis test. Spearman rank correlation analysis was used to analyze the correlation of perilipin family proteins expression in liposarcoma. Statistical significance was defined as *P < 0.05, **P < 0.01, ***P < 0.001.

## Results

### Perilipins expression in liposarcoma and normal adipose tissue

In total 66 liposarcoma cases showed different expression levels of perilipin family proteins. All five perilipins staining were detected in most liposarcoma cases (>50%). In 20 normal adipose tissue samples, PLIN1, PLIN2, PLIN4 and PLIN5 expressed in all cases (100%), while the expression rate of PLIN3 was 25% (5/20).

The positive rates of PLIN1 (P<0.001), PLIN2 (P=0.033), PLIN4 (P<0.001) and PLIN5 (P=0.033) were significantly decreased, compared with normal adipose tissues. However, the expression rate of PLIN3 (P=0.002) was obviously increased in liposarcoma (Table 1).

### The different expression of perilipins in various non-lipomatous sarcoma

**Figure 1:**
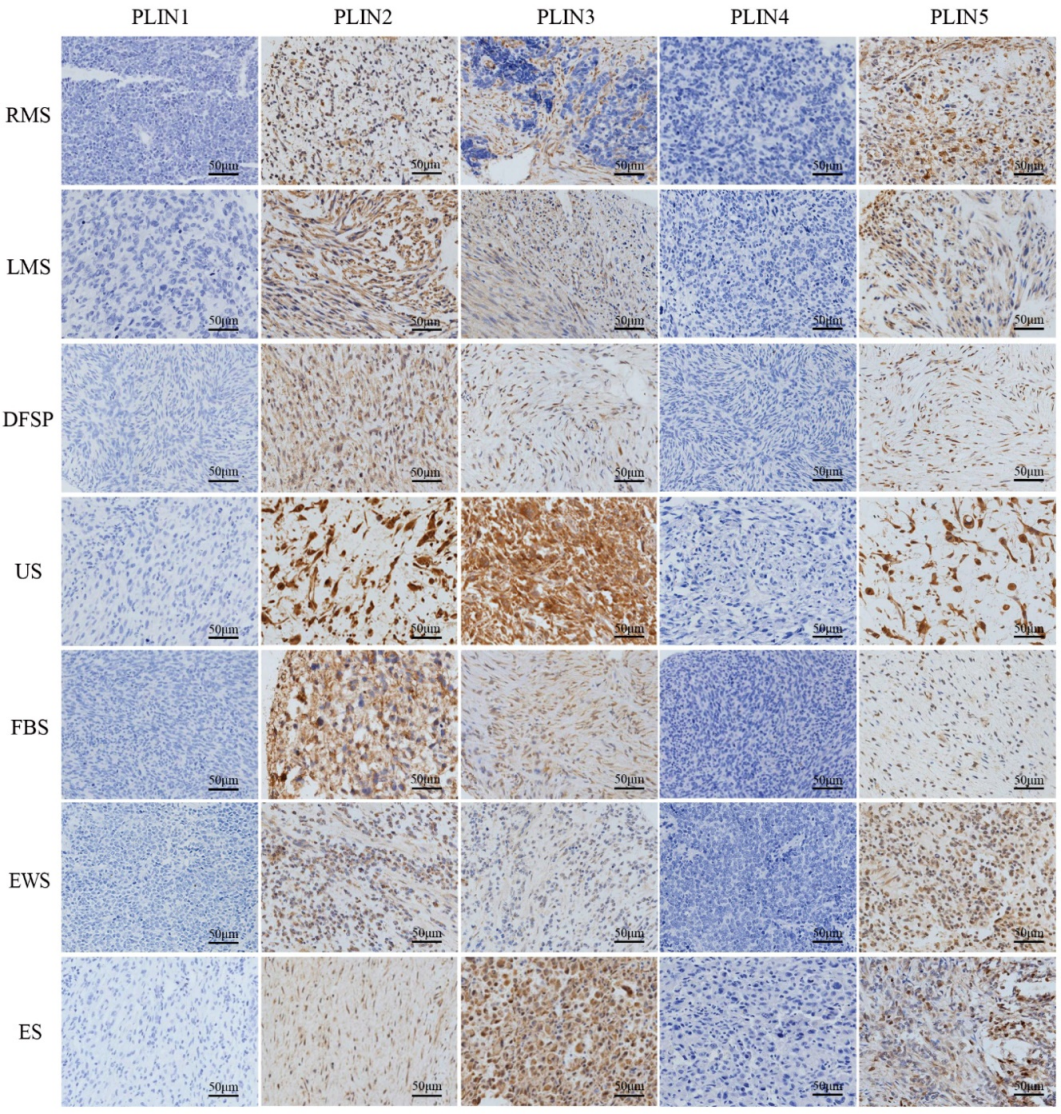
** Perilipins expression in seven non-lipomatous sarcomas.** PLIN1 and PLIN4 were completely lost after immunostaining was performed. PLIN2, PLIN3, and PLIN5 showed different positivity levels in the tumor cells. PLIN2 and PLIN5 exhibited cytoplasmic or membrane staining, and PLIN3 showed nuclear or cytoplasmic positivity. RMS, rhabdomyosarcoma; LMS, leiomyosarcoma; DFSP, dermatofibrosarcoma protuberans; US, undifferentiated sarcoma; FBS, fibrosarcoma. EWS. Ewing's sarcoma; ES. Epithelioid sarcoma

PLIN1 was absent from non-lipomatous sarcomas (0/179), PLIN2 expressed in all fibrosarcomas (18/18), Ewing's sarcomas (17/17), epithelioid sarcomas (8/8), and in most rhabdomyosarcomas (17/22), leiomyosarcomas (31/42), dermatofibrosarcoma protuberans (23/38), and undifferentiated sarcomas (33/34). PLIN3 expression was seen in all epithelioid sarcomas (8/8), in most dermatofibrosarcoma protuberans (31/38), undifferentiated sarcomas (25/34), fibrosarcomas (16/18), Ewing's sarcomas (11/17), and in a few rhabdomyosarcomas (5/22) and leiomyosarcomas (12/42). PLIN4 expression was negative in non-lipomatous sarcomas (0/171), except for epithelioid sarcomas (2/8). PLIN5 expressed in all epithelioid sarcomas (8/8), in more than half of undifferentiated sarcomas (27/34), fibrosarcomas (15/18), Ewing's sarcomas (13/17), rhabdomyosarcomas (14/22) and leiomyosarcomas (24/42), and in less than half of dermatofibrosarcoma protuberans (17/38). Kruskal-Wallis test analysis found that the expressions of PLIN2 (P<0.001), PLIN3 (P<0.001) and PLIN5 (P = 0.013) had a significant difference in various non-lipomatous sarcomas, except for epithelioid sarcomas (Table 2). As to the staining pattern on the cellular level, PLIN2, PLIN4 and PLIN5 exhibited cytoplasmic or membrane staining, and PLIN3 showed the nuclear or cytoplasmic staining pattern (Figure 1).

### Differential expression of perilipins in liposarcoma and non-lipomatous sarcoma

The results showed that the expression levels of PLIN1 (P<0.001) and PLIN4 (P<0.001) in liposarcoma were significantly higher than those in non-lipomatous sarcoma. However, the expression of PLIN2 (P=0.744), PLIN3 (P=0.117), and PLIN5 (P=0.062) showed no significant difference (Table 3).

### Perilipins expression in different subtypes of liposarcoma

Looking into four different subtypes among 66 liposarcoma cases, PLIN1 expression was seen in most WDLs (12/14), MLs (27/38), and in a few PLs (1/3) and DLs (1/11). The expression of PLIN1 increased with adipocytic differentiation of liposarcoma. PLIN2 expressed in all WDLs (14/14), PLs (3/3), and in more than half of MLs (30/38) and DLs (6/11). PLIN3 expression was shown in all PLs (3/3), and in most DLs (10/11), MLs (25/38), and WDLs (9/14). PLIN4 expressed in some well-differentiated liposarcomas, including most WDLs (11/14), and a few MLs (10/38), but was absent in DLs (0/11) and PLs (0/3). PLIN5 expression was seen in 92.86% of WDLs (13/14), 84.21% of MLs (32/38), 63.64% of DLs (7/11) and 33.33% of PLs (1/3). PLIN5 expression increased with adipocytic differentiation of liposarcoma.

**Figure 2:**
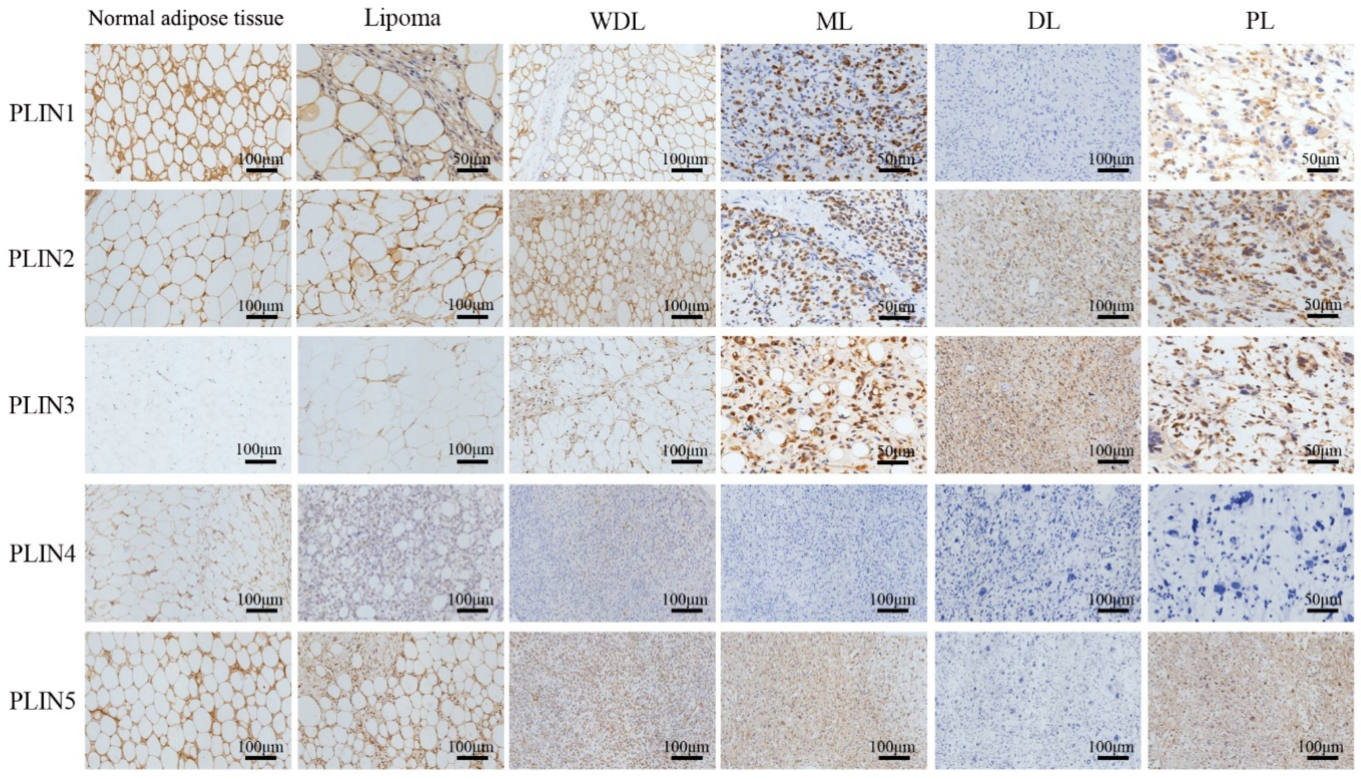
** Perilipins expression in different subtypes of liposarcoma.** Immunohistochemical staining revealed the different expression of five perilipins, PLIN1 exhibited strong membrane staining in benign tissues and well-differentiated sarcomas, and it showed different degrees of intensity in different subtypes of liposarcoma. PLIN4 showed faint membrane or cytoplasmic staining in fairly well-differentiated sarcomas and was absent in PL and DL. PLIN3 showed the nuclear or cytoplasmic staining pattern in high-grade sarcomas. PLIN2 and PLIN5 widely expressed in different subtypes of liposarcoma. WDL, well-differentiated liposarcoma; ML, myxoid liposarcoma; DL, dedifferentiated liposarcoma; PL, pleomorphic liposarcoma.

The expression rates of PLIN1 (P<0.001), PLIN2 (P=0.034), and PLIN4 (P<0.001) were significantly different in subtypes of liposarcoma, while the expression rates of PLIN3 (P=0.25) and PLIN5 (P=0.051) were not significantly different (Table 4). As to the staining pattern, PLIN1 exhibited membrane staining, PLIN2, PLIN4 and PLIN5 exhibited cytoplasmic or membrane staining, and PLIN3 showed the nuclear or cytoplasmic staining pattern (Figure 2).

### Correlation of perilipin family proteins expression in liposarcoma

**Figure 3:**
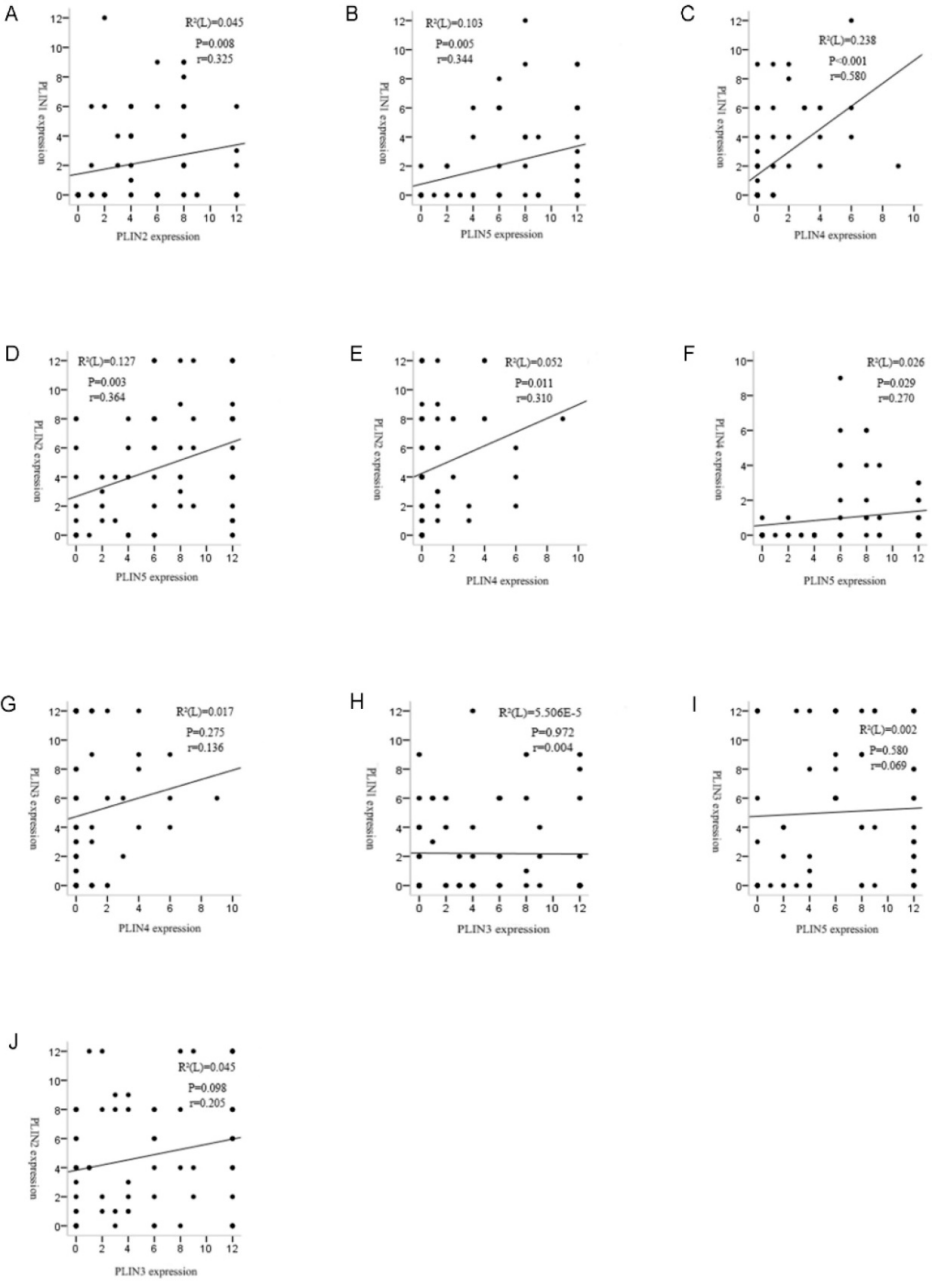
** Correlation analyses revealed strong relationships among the perilipins in liposarcoma except PLIN3.** (A) Significant correlation was observed between the expression of PLIN1 and PLIN2 in liposarcoma (r = 0.325, P = 0.008). (B) Significant correlation between the expression of PLIN1 and PLIN5 in liposarcoma (r = 0.344, P = 0.005). (C) Significant correlation was observed between the expression of PLIN1 and PLIN4 in liposarcoma (r = 0.580, P = 0.000). (D) Significant correlation between the expression of PLIN2 and PLIN5 in liposarcoma (r = 0.364, P = 0.003). (E) Significant correlation was observed between the expression of PLIN2 and PLIN4 in liposarcoma (r = 0.310, P = 0.011). (F) Significant correlation between the expression of PLIN4 and PLIN5 in liposarcoma (r = 0.270, P = 0.029). (G, H, I and J) There were no correlations between PLIN3 and the other four proteins.

We investigated the relationship between the expressions of perilipin members. Spearman's rank analysis demonstrated positive correlation between PLIN1 and PLIN2 (r =0.325, P = 0.008), PLIN1 and PLIN4 (r =0.580, P = 0.000), PLIN1 and PLIN5 (r =0.344, P = 0.005), PLIN2 and PLIN4 (r =0.310, P = 0.011), PLIN2 and PLIN5 (r =0.364, P = 0.003), PLIN4 and PLIN5 (r =0.270, P = 0.029) expressions in liposarcoma, respectively. However, PLIN3 was not shown to be associated with the other four proteins (Figure 3).

### Perilipins expression and clinical pathological parameters of liposarcoma

The study included 66 patients with liposarcoma, comprising 26 males and 40 females. Of these patients, 53 were of Han ethnicities, and 13 belonged to ethnic minorities (including Uygur and Kazakh). The average age of patients was 58.65 years. The youngest patient was 2 years old, and the oldest was 89 years old. Our results revealed 2 cases of head and neck, 42 cases of extremities, 16 cases of chest and abdomen, and 6 cases of other parts.

The patients with liposarcoma were grouped on the basis of gender, age, ethnicity, tumor location, size, and tissue type. The expression levels of PLIN1 (P<0.001), PLIN2 (P<0.05), PLIN3 (P<0.05), and PLIN5 (P<0.001) were closely related to the histological type of liposarcoma. However, these proteins were not related to the patients' gender, age, ethnicity, tumor location, or size (P>0.05) (Table 5).

## Discussion

Perilipin family proteins are widely expressed in hepatocytes, skeletal muscle cells, macrophages, endothelial cells, fibroblasts, adipocytes and myoblasts 24-26. Perilipins are closely related to the function of lipid droplets, and their abnormal expressions are associated with many diseases, especially lipid metabolic diseases. PLIN2 specifically expresses in atherosclerotic plaques 27, 28. Lacking PLIN1 can cause hypertrophic cardiomyopathy 29. High expression of PLIN5 leads to myocardial lipid accumulation and cardiomyopathy caused by type 1 diabetes 30. Furthermore, the different expression of perilipins has become a sensitive indicator of parotid gland carcinoma 19, early rectal cancer 20, lung adenocarcinoma 31 and cervical cancer 32.

The difficulty in the diagnosis of liposarcoma is that it is hard to distinguish tumor cells or fat vacuoles, similar to adipocyte differentiation from adipocytes. Fat staining is difficult to apply to paraffin samples to distinguish fat vacuoles from degenerative or absorbable fat vacuoles. In lipid droplets formation, perilipins were not produced at the same time. For example, PLIN1 forms when mature lipid droplets begin to form and then gradually increases until it is coated on the surface of mature lipid droplets at high levels 33-35. By contrast, PLIN4 is rich at the initial stage of small fat droplets and gradually reduces as small lipid droplets become mature 36, 37. Interestingly, the expression levels of PLIN1 and PLIN4 in liposarcoma and non-lipomatous sarcoma had similar results which showed almost no expression in non-lipomatous sarcoma. Westhoff CC et al. have reported differential expression of PLIN1 in liposarcoma and non-liposarcoma 21, and Straub BK et al. also found that PLIN4 expression was similar to PLIN1 in adipocytic tumors 22. Our study further confirms this finding in a larger cohort and in more non-lipomatous sarcoma types. It suggests that PLIN1 and PLIN4 were more important in identifying liposarcoma and non-lipomatous sarcoma that contained some specific overlapping diagnostic cells.

The expression of perilipins also varies in each subtype of liposarcoma. Our study showed that the differences in the expression levels of PLIN1, PLIN2, and PLIN4 in various subtypes were statistically significant (P<0.005). It is worth noting that the expression of PLIN1 and PLIN4 in various subtypes of liposarcoma are still similar. A previous study has shown that PLIN2 was significantly higher in sebaceous cancer than in other non-adenocarcinoma tissues of skin tumors and helped distinguish adenocarcinoma and other tumor tissues in overlapping tissues 38. PLIN2 was downregulated during the differentiation of pre-adipocytes to adipocytes and was undetected in mature adipocytes 39. In our study, we found that the positive expression rate of PLIN2 in various types of liposarcomas decreased with the degree of differentiation, but PLIN2 showed strong positive expression in three PL cases and the research by Westhoff CC et al. also showed high PLIN2 expression level in PL 21. This finding might be related to the obviously atypical nature of PL and might contribute to the diagnosis and treatment of PL. However, it should be verified because of the small sample size of PL.

Studies have shown the increase of PLIN1 expression with adipocytic differentiation of liposarcoma^21^. As the dedifferentiation of DL could occur at any stage of WDL recurrence, the opposite expression in DL of PLIN1 and PLIN3 confirmed that PLIN1 expressed in mature LDs, that is, well-differentiated fat cells, while PLIN3 was mainly found in small LDs. As LDs matured, PLIN3 gradually decreased. Therefore, PLIN3 was highly expressed in poorly differentiated DL, whereas the expression of PLIN1 was low. Therefore, whether the expression of RNA levels is different can be verified to determine whether PLIN1 and PLIN3 could be potential molecular targets of DL.

PLIN3 is a blood marker of cervical cancer and biomarker of cervical developmental disorders and invasive cancers 32. Studies have shown that the growth of cervical cancer cells with knocked-out PLIN3 was inhibited. PLIN3 expression is related to the aggregation of triglycerides, and PLIN3 leads to the reduction of triglyceride aggregation and the inhibition of LD maturity 40. Its expression in liposarcoma is higher than that in normal adipose tissue and may be related to female hormones and blood levels 41. In various types of liposarcoma, the poorer the differentiation was, the higher the degree of malignancy and the higher the expression of the PLIN3 protein would be, except in ML. Therefore, this might suggest that we could further verify the possible relationship between the development of PLIN3 and ML to determine whether PLIN3 has a labeling meaning.

Although perilipins expression have been reported in different mesenchymal tumors^22^, there are fewer tumors involving malignant sarcomas and no more specific analysis has been performed. We selected seven typical non-liposarcomas to observe the expression of perilipins. However, due to the small sample size of epithelioid sarcomas, we analyzed the expression of perilipins in the remaining six sarcomas and found that PLIN2, PLIN3 and PLIN5 showed different and rich expressions in non-lipomatous sarcomas, especially high-grade sarcoma. In contrast, the staining of PLIN1 and PLIN4 almost completely disappeared. It also reminds us that while other molecules are difficult to use to identify non-lipomatous sarcomas, can we observe the degree of differentiation by PLIN2, PLIN3 and PLIN5 staining to help diagnosis. This still needs to be verified by expanding the sample size.

By analyzing the correlation of perilipin family proteins expressions in liposarcoma, we found that apart from PLIN3, the other four proteins' expression had significant correlations with each other. It can be noted that this shows some similar results in the analyses of the various subtypes of liposarcoma, which suggested an obviously increased expression rate of PLIN3 in liposarcoma compared with the normal control group, while the other four proteins' expression were shown to be significantly reduced in liposarcoma. This may indicate that PLIN3 might play a different role with the other four molecules in the development of liposarcoma. For the study, we have used both TMAs and the whole tumor slides. Since heterogeneity is typical for sarcoma, we also compared the positive rates of the two sample types, and there is no much impact on the results.

In general, PLIN1 and PLIN4 might be of potential use as ancillary diagnostic indicators in liposarcoma. PLIN1 could be suitable for discriminating differentiated degrees of liposarcoma. Further work is required in assessing perilipins' expression at cellular level, and particularly required to further explore the role of perilipins in the development of liposarcoma.

## Supplementary Material

Supplementary figure.

## Figures and Tables

**Table 1:** Immunohistochemical expression of the perilipins in liposarcoma and normal adipose tissue

	liposarcoma	normal adipose tissue	P
	n = 66 (%)	n = 20 (%)	
PLIN1	41 (62.12%)	20 (100%)	< 0.001
PLIN2	53 (80.30%)	20 (100%)	0.033
PLIN3	47 (71.21%)	5 (25.00%)	0.002
PLIN4	21 (31.82%)	20 (100%)	< 0.001
PLIN5	53 (80.30%)	20 (100%)	0.033

*P < 0.05, **P < 0.01, ***P < 0.001.

**Table 2:** Immunohistochemical expression of the perilipins in various non-lipomatous sarcomas

	RMS n=22 (%)	LMS n=42 (%)	DFSP n=38 (%)	US n=34 (%)	FBS n=18 (%)	EWS n=17 (%)	ES n=8 (%)	P
PLIN1								1
-	22(100.0)	42(100.0)	38(100.0)	34(100.0)	18(100.0)	17(100.0)	8 (100.0)	
+	0 (00.00)	0 (00.00)	0 (00.00)	0 (00.00)	0 (00.00)	0 (00.00)	0 (00.00)	
++	0 (00.00)	0 (00.00)	0 (00.00)	0 (00.00)	0 (00.00)	0 (00.00)	0 (00.00)	
+++	0 (00.00)	0 (00.00)	0 (00.00)	0 (00.00)	0 (00.00)	0 (00.00)	0 (00.00)	
PLIN2								<0.001
-	5(22.73)	11(26.19)	15(39.47)	1(2.94)	0 (00.00)	0 (00.00)	0(00.00)	
+	15(68.18)	13(30.95)	13(34.21)	15(44.12)	6 (33.33)	16(94.12)	5(62.50)	
++	2(9.09)	15(35.71)	4(10.53)	12(35.29)	10(55.56)	1(5.82)	3(37.50)	
+++	0 (00.00)	3(7.14)	6(15.79)	6(17.65)	2(11.11)	0 (00.00)	0 (00.00)	
PLIN3								<0.001
-	17(77.27)	30(71.43)	7(18.42)	9(26.47)	2(11.11)	6(35.29)	0(00.00)	
+	5(22.73)	9(21.43)	20(52.63)	10(29.41)	14(77.78)	11(64.71)	0(00.00)	
++	0 (00.00)	1(2.38)	3(7.90)	5(14.71)	2(11.11)	0 (00.00)	1(12.50)	
+++	0 (00.00)	2(4.76)	8(21.05)	10(29.41)	0 (00.00)	0 (00.00)	7(87.50)	
PLIN4								1
-	22(100.0)	42(100.0)	38(100.0)	34(100.0)	18(100.0)	17(100.0)	6(75.00)	
+	0 (00.00)	0 (00.00)	0 (00.00)	0 (00.00)	0 (00.00)	0 (00.00)	1(12.50)	
++	0 (00.00)	0 (00.00)	0 (00.00)	0 (00.00)	0 (00.00)	0 (00.00)	1(12.50)	
+++	0 (00.00)	0 (00.00)	0 (00.00)	0 (00.00)	0 (00.00)	0 (00.00)	0(00.00)	
PLIN5								0.013
-	8(36.36)	18(42.86)	21(50.00)	7(20.59)	3(16.67)	4(23.53)	0(00.00)	
+	6(27.27)	17(40.48)	9(21.05)	14(41.18)	10(55.56)	9(52.94)	5(62.50)	
++	3(13.64)	6(14.29)	8(7.90)	5(14.71)	5(27.78)	4(23.53)	3(37.50)	
+++	5(22.73)	1(2.38)	0(21.05)	8(23.53)	0 (00.00)	0(00.00)	0(00.00)	

*Due to the small sample size of PL may cause bias, we included the remaining six tumors for statistical analysis. RMS. Rhabdomyosarcoma; LMS. Leiomyosarcoma; DFSP. Dermatofibrosarcoma protuberans; US. Undifferentiated sarcoma; FBS. Fibrosarcoma; EWS. Ewing's sarcoma; ES. Epithelioid sarcoma; *P < 0.05, **P < 0.01, ***P < 0.001.

**Table 3:** Immunohistochemical expression of the perilipins in liposarcoma and non-lipomatous sarcomas

	liposarcoma	non-lipomatous sarcomas	P
	n=66(%)	n=179(%)	
PLIN1			<0.001
-	25 (37.88)	179 (100.0)	
+	10(15.15)	0 (00.00)	
++	24 (36.36)	0 (00.00)	
+++	7 (10.61)	0 (00.00)	
PLIN2			0.744
-	13(19.70)	32(17.88)	
+	21(31.82)	83(46.37)	
++	17(25.76)	47(26.26)	
+++	15(22.73)	17(9.50)	
PLIN3			0.117
-	19(28.79)	71(39.65)	
+	24(36.36)	69(38.55)	
++	14(21.21)	12(6.70)	
+++	9(13.64)	27(15.08)	
PLIN4			<0.001
-	45(68.18)	177(98.88)	
+	13(19.70)	2(1.12)	
++	7(10.61)	0(00.00)	
+++	1(1.52)	0(00.00)	
PLIN5			0.062
-	13(19.70)	57(31.84)	
+	25(37.88)	74(41.34)	
++	13(19.70)	34(18.99)	
+++	15(22.73)	14(7.82)	

*P < 0.05, **P < 0.01, ***P < 0.001.

**Table 4:** Immunohistochemical expression of the perilipins in subtypes of liposarcoma

	WDL/ALT n=14 (%)	ML n=38 (%)	DL n=11 (%)	PL n=3 (%)	P
PLIN1					<0.001
-	2 (14.29)	11(28.95)	10(90.91)	2(66.67)	
+	1 (7.14)	7 (18.42)	1 (9.09)	1(33.33)	
++	8 (57.14)	16 (42.11)	0(00.00)	0(00.00)	
+++	3 (21.43)	4 (10.53)	0(00.00)	0(00.00)	
PLIN2					0.034
-	0(00.00)	8(21.05)	5(45.46)	0(00.00)	
+	3(21.43)	12(31.58)	4(36.36)	1(33.33)	
++	6(42.86)	8(21.05)	2(18.18)	2(66.67)	
+++	5(35.71)	10(26.32)	0(00.00)	0 (00.00)	
PLIN3					0.25
-	5(35.71)	13(34.21)	1(9.09)	0(00.00)	
+	6(42.86)	15(39.47)	2(18.18)	1(33.33)	
++	3(21.43)	5(13.16)	4(36.36)	2(66.67)	
+++	0 (00.00)	5(13.16)	4(36.36)	0(100.0)	
PLIN4					<0.001
-	3(21.43)	28(73.68)	11(100.00)	3(100.0)	
+	9(64.29)	4(10.53)	0(00.00)	0(00.00)	
++	2(14.29)	5(13.51)	0(00.00)	0(00.00)	
+++	0(00.00)	1(2.63)	0(00.00)	0(00.00)	
PLIN5					0.051
-	1(7.14)	6(15.79)	4(36.36)	2(66.67)	
+	1(7.14)	18(47.37)	5(45.46)	1(33.33)	
++	7(50.00)	5(13.16)	1(9.09)	0(00.00)	
+++	5(35.71)	9(23.68)	1(9.09)	0(00.00)	

WDL: Well-differentiated liposarcoma; ALT: Atypical lipomatous tumor; ML: Myxoid liposarcoma; DL: Dedifferentiated liposarcoma; PL: Pleomorphic liposarcoma. *P < 0.05, **P < 0.01, ***P < 0.001.

**Table 5:** Relationship between Perilipins family protein expression and clinicopathological parameters in patients with liposarcoma

Clinical pathological parameters	n	PLIN1			PLIN2			PLIN3			PLIN4			PLIN5	
			
		+	P		+	P		+	P		+	P		+	P
Sex															
Male	26	16(61.5)			19(73.1)			12(46.2)			3(11.5)			15(57.7)	
Female	40	15(37.5)	P=0.056		20(50.0)	P=0.062		11(27.5)	P=0.12		6(15.0)	P=0.689		14(35.0)	P=0.07
Age(y)															
≥50	45	18(40.0)			23(51.1)			18(40.0)			6(13.3)			18(40.0)	
<50	21	13(61.9)	P=0.097		9(42.9)	P=0.532		13(61.9)	P=0.199		3(14.3)	P=0.916		10(47.6)	P=0.56
Ethnos															
Han	53	24(45.3)			26(49.1)			18(34.0)			8(15.1)			22(41.5)	
Others	13	7(53.8)	P=0.579		6(46.2)	P=0.851		5(38.5)	P=0.76		1(7.7)	P=0.675		6(46.2)	P=0.761
Tumor size															
≥5cm	55	29(52.7)			27(49.1)			19(34.5)			7(12.7)			25(45.5)	
<5cm	11	3 (27.3)	P=0.123		5(45.5)	P=0.826		4(36.4)	P=0.908		2(18.2)	P=0.63		3(27.3)	P=0.265
Location															
Head and neck	2	2(100.0)			2(100.0)			1(50.0)			0(0.0)			2(100.0)	
Limb and trunk	42	22(52.4)			19(45.2)			12(28.6)			5(11.9)			14(33.3)	
Posterior abdomen	16	5(31.3)	P=0.212		8(50.0)	P=0.639		8(50.0)	P=0.419		3(18.8)	P=0.731		8(50.0)	P=0.102
Others	6	2(33.3)			3(50.0)			2(33.3)			1(16.7)			4(66.7)	

## Abbreviations

ALT: atypical liposarcoma
WDL: well-differentiated liposarcoma
DL: dedifferentiated liposarcoma
ML/RCL: myxoid/round cell liposarcoma
PL: pleomorphic liposarcoma
PLIN1: perilipin
PLIN2: adipophilin
PLIN3: tail-interacting protein of 47kDa
PLIN4: plasma membrane associated protein
PLIN5: oxidative PAT
TMA: Tissue microarray
IHC: Immunohistochemistry
PBS: phosphate-buffered saline
