# Supplementary material for: The Expression of Perilipin Family Proteins can be used as Diagnostic Markers of Liposarcoma and to Differentiate Subtypes
Source: J Cancer. 2020 Apr 7;11(14):4081–90. doi: 10.7150/jca.41736 (PMC7196260; doi:10.7150/jca.41736)
Supplement: Supplementary file 1 — Supplementary figure. [file jcav11p4081s1.pdf]

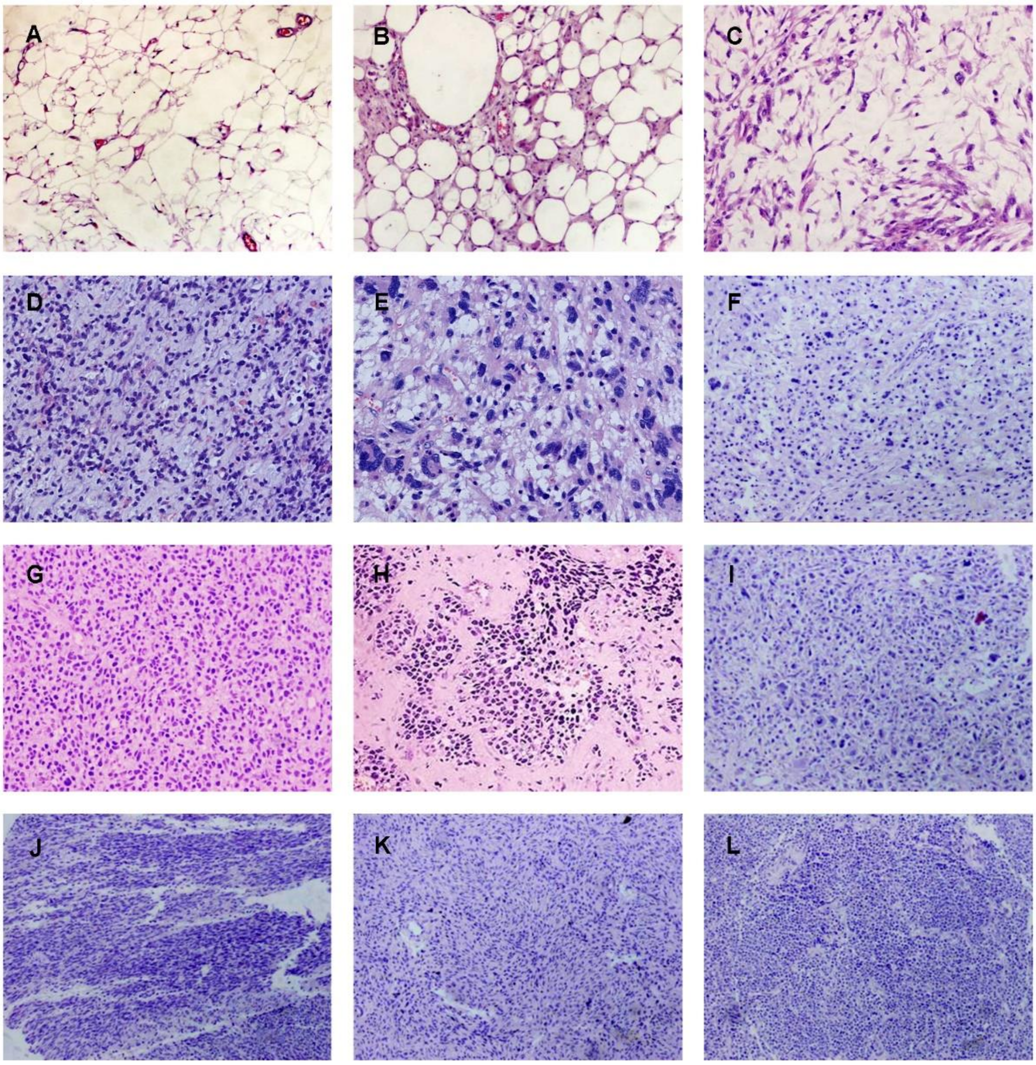

Haematoxylin and eosin-stained (H&E) morphology of the disease used in our study. Normal adipose tissue (A); Well-differentiated liposarcoma (B); Myxoid liposarcoma (C); Dedifferentiated liposarcoma (D); Pleomorphic liposarcoma (E); fibrosarcoma (F); Embryonal rhabdomyosarcoma (G); Alveolar rhabdomyosarcoma (H); leiomyosarcoma (I); Dermatofibrosarcoma protuberans (J); undifferentiated sarcoma (K); Ewing’s sarcoma (L).
